# hUMSC transplantation restores follicle development in ovary damaged mice via re-establish extracellular matrix (ECM) components

**DOI:** 10.1186/s13048-023-01217-y

**Published:** 2023-08-24

**Authors:** Yin Shuyuan, Wang Meimei, Li Fenghua, Zhao Huishan, Chu Min, Bao Hongchu, Liu Xuemei

**Affiliations:** Reproductive Medicine Centre, The Affiliated Yantai Yuhuangding Hospital of Qingdao University, Yantai, 264000 Shandong China

**Keywords:** Follicular Development, Ovarian Reserve, Transcriptome sequencing, Extracellular matrix, Mesenchymal stem cells

## Abstract

**Objectives:**

Explore the therapeutic role of human umbilical mesenchymal stem cells (hUMSCs) transplantation for regeneration of ECM components and restoration of follicular development in mice.

**Background:**

The extracellular matrix (ECM) is crucial to maintain ovary function and regulate follicular development, as it participates in important cell signaling and provides physical support to the cells. However, it is unknown how hUMSCs affect the expression of ECM-related genes in ovaries treated with cyclophosphamide (CTX) and busulfan (BUS).

**Methods:**

In the present study, we used 64 six- to eight-week-old ICR female mice to established mouse model. The mice were randomly divided into four groups (n = 16/group): control, POI, POI + hUMSCs, and POI + PBS group. The premature ovarian insufficiency (POI) mouse model was established by intraperitoneal injection of CTX and BUS for 7days, then, hUMSCs or PBS were respectively injected via the tail vein in POI + hUMSCs or POI + PBS group. Another 7days after injection, the mice were sacrificed to harvest the ovary tissue. The ovaries were immediately frozen with liquid nitrogen or fixed with 4% PFA for subsequent experiments. To screen differentially expressed genes (DEGs), we performed transcriptome sequencing of ovaries. Thereafter, a Gene Ontology (GO) terms and Kyoto Encyclopedia of Genes and Genomes (KEGG) pathway analyses were performed to predict the related biological functions. Retrieval of interacting genes for ECM-related DEGs was performed using the function of STRINGdb (version 2.6.5) to evaluate potential protein-protein interaction (PPI) networks. Furthermore, qRT-PCR and IHC were performed to assess the differential expression of selected DEGs in control, damaged, hUMSCs-transplanted and non-transplanted ovaries.

**Results:**

Chemotherapy caused mouse ovarian follicular reserve depletion, and hUMSCs transplantation partially restored follicular development. Our results revealed that ECM-receptor interaction and ECM organization were both downregulated in the damaged ovaries. Further investigation showed that ECM-related genes were downregulated in the CTX and BUS treatment group and partially rescued in hUMSCs injection group but not in the PBS group. qRT-PCR and IHC verified the results: collagen IV and laminin gamma 3 were both expressed around follicle regions in normal ovaries, chemotherapy treatment disrupted their expression, and hUMSCs transplantation rescued their localization and expression to some extent.

**Conclusion:**

Our data demonstrated that ECM-related genes participate in the regulation of ovarian reserve, hUMSCs treatment rescued abnormal expression and localization of collagen IV and laminin gamma 3 in the damaged ovaries. The results suggest that hUMSCs transplantation can maintain ECM-stable microenvironments, which is beneficial to follicular development.

**Supplementary Information:**

The online version contains supplementary material available at 10.1186/s13048-023-01217-y.

## Introduction

The ECM is composed of proteins, proteoglycans, and glycosaminoglycans (GAGs). ECM-stable microenvironments favor cell homeostasis, whereas ECM structural abnormalities can cause functional deficits and fibrogenesis [1–3].

ECM is required for ovarian development and participates in folliculogenesis and the generation of ovarian epithelial cells. There are many primordial follicles in the ovarian epithelium, and collagen provides structural support for the primordial follicle pool [4]. ECM provides suitable rigidity for dormant primordial follicles in the ovarian cortical tissue [5–7]. When follicle activation occurs, the activated follicles migrate to ovarian medulla tissue, which is softer than the cortex tissue and beneficial for follicular development [8]. Starting from adolescence, a few follicles are activated and can develop into mature follicles and become ovulated during every menstrual cycle [9].

To date, there have been many studies on follicular development and ovulation and the associated regulation of hormone signaling and molecular mechanisms. However, few studies have focused on the effects of ECM on follicular development. The syntheses of ECM proteins in adult ovaries were significantly greater than those in perimenopausal and menopausal ovaries [10]. The content of ECM proteins, such as collagen [11], laminin [11] and elastin [12] all increased in the early embryonic developmental stage, providing appropriate stiffness for growth and development. Stable ECM expression supports ovary morphology and promotes ovulation. Abnormal ECM may alter ovarian elasticity and cause ovarian stiffness or fibrosis, affecting ovarian follicle development and ovulation [13].

The primordial follicles go through the primary follicle, secondary follicle, preantral follicle and antral follicle stages until ovulation. After the luteinizing hormone (LH) surge, antral follicles with a certain diameter enlarge, bulge out the ovarian surface epithelium and ovulate [14]. Briefly, the top of the preovulatory follicle is constructed with a layer of epithelial cells and collagen. The ECM not only provides structural support for the follicle but also maintains cellular connectivity and regulates a wide variety of biological processes including cell proliferation, development, differentiation, migration and metabolism [15].

Collagens are major constituents of the ECM in ovarian tissue [16]. Ouni et al. further studied the expression of elastic matrisome components, such as collagen, elastin, fibrillin-1 and GAGs, in the ovaries of prepubescent girls, reproductive age women and postmenopausal women and further analysed the components of the ECM surrounding preantral follicles at pre- and late-pubertal stages. They concluded that ECM is essential the follicular development and homeostasis [10]. With respect to follicular growth, studies have demonstrated increased laminin expression and have shown that laminin and fibronectin harbor binding sites for integrin, which can initiate intracellular signaling cascades leading to enhanced cell proliferation and differentiation [17].

In growing follicles, the communications between granulosa cells (GCs) and theca cells (TCs), GCs and GCs, and GCs and oocytes provide pathways for the exchange of small molecules and nutrients. TCs are critical for follicular growth, and can produce sexual steroid hormones at antral follicles in vitro, indicating crucial roles particularly in gonadotropin independent phases [18, 19]. Collagen IV is localized at the ovarian TC region and stroma in mice and its expression is initiated in primary follicles and is increased in antral follicles. Laminin is localized the at ovarian TC region, stroma and ovarian surface epithelium, especially around TCs. The above two proteins’ conspicuous staining encircles follicles [20]. Studies have also shown that the expression of Fbn1 (fibrillin 1) decreases from adolescence to menopause and that Fbn3 (fibrillin 3) is highly expressed during stromal expansion before the follicle formation stage, suggesting that Fbns are essential for ovarian and follicular development [10, 21].

ECM homeostasis plays a critical role in maintaining normal follicular development, and ECM component expression that is either too high or too low can have adverse effects on ovarian function. Genetic, autoimmune iatrogenic, and environmental factors, etc., can trigger ovarian hypofunction and deplete the primordial follicle pool. In addition, the use of chemotherapeutic drugs in cancer patients may induce damage to ovarian function, such as follicular atresia [22], cortical fibrosis [23], oocyte apoptosis [24], impaired GC function [25] and abnormal hormone secretion [26]. MSCs exist in various tissues, such as adipose, bone marrow, placenta and umbilical cord, and they can secrete a wide range of antioxidant, anti-fibrosis, anti-apoptotic and anti-inflammatory cytokines [27]. It has been reported that MSCs have beneficial effects on ovarian damage repair and they can also regulate hormone secretion, decrease GC apoptosis, increase follicle quantity and quality, improve ovarian reserve, and play other roles in improving ovarian function, thereby alleviating the side effects of chemotherapy [26, 28, 29].

The aim of this study was to decipher the therapeutic role of hUMSCs transplantation on damaged ECM components in mouse ovaries. We focused on ECM organization through transcriptome sequencing and performed both transcriptional and protein analyses. IHC results demonstrated the abnormal expression and localization of collagen IV and laminin gamma 3 in the damaged ovaries, whereas hUMSCs treatment rescued their expressions. This study illustrated that ECM-stable microenvironments were critical for follicular development, and hUMSCs transplantation was effective in restoring chemotherapy-induced ovarian damage.

## Materials and methods

### Animals

Six- to eight-week-old ICR female mice were purchased from the Jinan Pengyue Experimental Animal Breeding, Co., Ltd. (Shandong, China). The mice were housed in a temperature-controlled room with a 12 h/12 hr light-dark cycle, and fed a regular diet. All procedures with mice were conducted according to the rules stipulated by the Animal Care and Use Committee of Yantai Yuhuangding Hospital.

### hUMSC culture and characterization

The hUMSCs were kindly provided by Shandong Qilu Stem Cell Engineering Co., Ltd. The cells were cultured in serum-free medium according to standard experimental protocols approved by the provider. Passage 4 hUMSCs were collected for tail vein injection.

### Establishment of the primary ovarian insufficiency (POI) model

Female ICR mice were treated with CTX (120 mg/kg, dissolved in saline) and BUS (20 mg/kg, dissolved in DMSO) to generate the POI model. Total 64 mice were randomly divided into four groups (n = 16/group): control, POI, POI + hUMSCs and POI + PBS. In the POI group, the mice were injected intraperitoneally with a 120 mg/kg CTX and 20 mg/kg BUS mixture. Controls were injected intraperitoneally with an equal volume of solvent at the same time. In the POI + hUMSCs group, 7 days after the injection of CTX and BUS, 1 × 10^6^ hUMSCs diluted in 100 µl PBS were injected into the tail vein. For the POI + PBS group, POI mice were injected with 100 µl of PBS via the tail vein. Another 7 days after the injection of hUMSCs or PBS, the mice were sacrificed for the following studies.

### Hematoxylin and eosin (HE) staining and ovarian follicle counting

The ovarian tissues were collected and washed at least 3 times with cold PBS and fixed in 4% paraformaldehyde (PFA) for at least 24 h. After dehydration and paraffin embedding, the samples were processed by sectioning (5 μm) and HE staining. To analyse the numbers of ovarian follicles, primordial, primary, secondary and antral follicles were classified and counted as described previously [30].

### RNA-seq data processing

RNA-seq raw reads were trimmed to remove adapters and low-quality reads using TrimGalore (version 0.6.6) with the parameters “-q 25 --phred33 --stringency 3 --length 36 -e 0.1”. These processed reads were then aligned to the mouse reference genome (Ensemble mm10) using STAR (version 2.7.3) with the default parameters. Mapped reads with high confidence were kept for further analysis using SAMtools (version 1.9). Expression levels for all Refseq genes were quantified to transcripts per kilobase of exon model per million mapped reads (TPM)using StringTie (version 2.1.4), and TPM values of replicates were averaged. To perform differential gene expression analysis, we first calculated the read counts of each RNA-seq sample using FeatureCounts (version 2.0.0). Then, we used Deseq2 in R to perform differential analysis. Genes with p ≤ 0.05 and FC > 1.2 or FC < -1.2 were defined as differentially expressed. DEG functional enrichment was analysed using online tools in Metascape (https://metascape.org/). The most significant GO terms and KEGG pathways were selected to visualize in R. To evaluate potential PPIs, DEGs were mapped in R using the function of STRINGdb (version 2.6.5) for the Retrieval of Interacting Genes under the default settings; node connections less than 5 were excluded. The PPI network of 23 downregulated ECM genes was visualized in R.

### Real-time reverse transcription polymerase chain reaction

Reverse transcription was conducted using HiScript III RT SuperMix for qPCR (+ gDNA wiper) (Vazyme, R323-01) following the manufacturer’s instructions. Real-time PCR was performed using 2 × SYBR Green qPCR Mix (with ROX) (SparkJade, AH0104). Data are shown as the fold change = 2^-ΔΔCt^ mean ± s.d. The primers are listed in Table S1.

### Immunohistochemistry

The expression of Collagen IV, Laminin gamma3 and Fibrillin 2 within ovarian tissues were detected by immunohistochemistry staining on the paraffin slide respectively. The ovarian sections fixed on the paraffin performed dewaxing and rehydration, antigen retrieval, and incubated in wet box with 3% H_2_O_2_ for 10 min sequentially. After blocking with 3% BSA, the slides were then immunostained with primary antibodies of Collagen IV (1:400, Abcam, ab236640), Laminin gamma3 (1:200, Abcam, ab234429) and Fibrillin 2 (1:200, Proteintech, 20252-1-AP) in a humidified box at 4 ℃ overnight. The slides were washed three times, and incubated with secondary antibody anti-rabbit (1:500, Sangon Biotech, D110065) at 37 °C for 30 min. Then the slides were stained with diaminobenzidine (DAB) as chromogen and counterstained with hematoxylin according to manufacturer’s instruction. After dehydration, the coverslips were mounted, and immunohistochemical images were taken using Leica Camera (Leica Microsystem, Buffalo Grove, IL, United States). Protein expressions were quantified with Image J software.

### Statistical analysis

Statistical analyses were performed in R (www.r-project.org/) and GraphPad Prism software. Statistical significance was calculated with One-way ANOVA with Tukey test (*P* < 0.05 was considered statistically significant). All experiments were repeated at least three times unless otherwise stated. The results are expressed as the mean ± s.d. Differences are shown with *(*P* < 0.05) and **(*P* < 0.01).

## Results

### hUMSCs transplantation restored follicle development in CTX-and BUS- treated mice

**Fig. 1:**
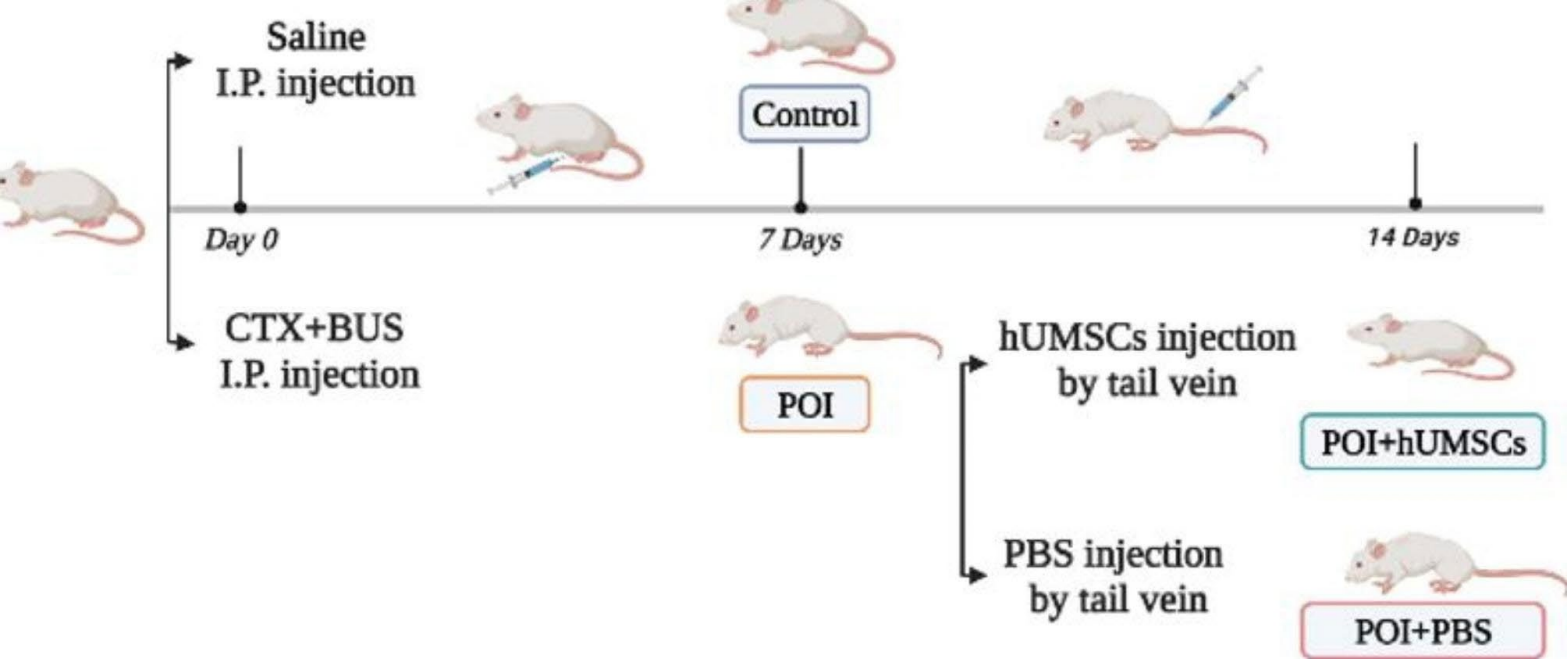
Schematic presentation of the POI animal model construction and hUMSCs treatment

**Fig. 2:**
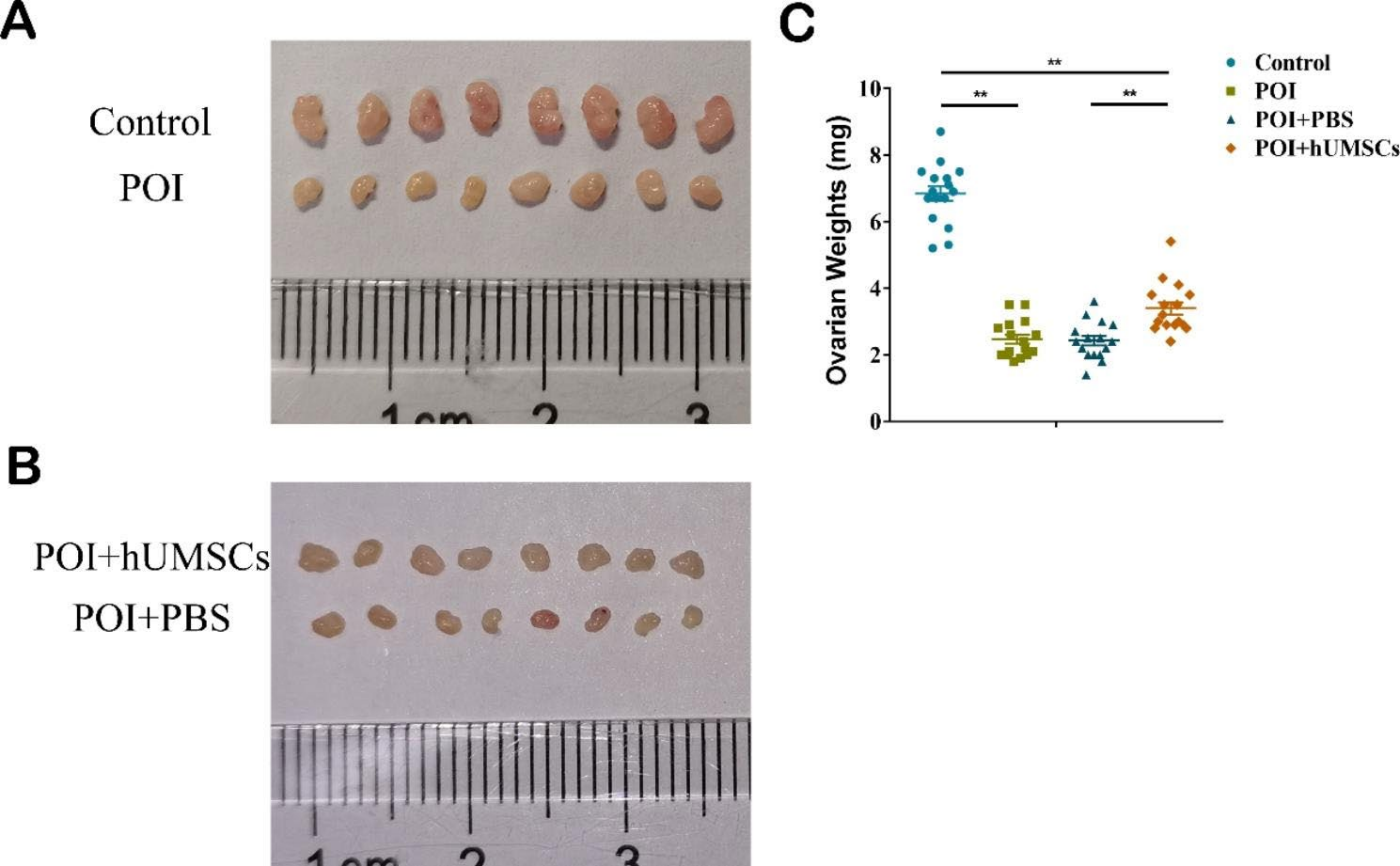
Morphological features and weights of the ovary. **(A)** Morphology of ovaries in Control, POI, POI + hUMSCs and POI + PBS groups. **(B)** Ovarian weights in Control, POI, POI + hUMSCs and POI + PBS groups (n = 16). **P < 0.01

To investigate the effects of hUMSCs transplantation on follicle development, we established a CTX + BUS-induced POI mouse model, as shown in Fig. 1. The ovaries in the POI and POI + PBS groups exhibited more atrophic phenotypes and lighter weights than those in the control and POI + hUMSCs groups (Fig. 2A and B). The weights of ovaries in the POI group were decreased to approximately 36% (2.463 ± 0.1732 vs. 6.844 ± 0.2282, n = 16, *P* ＜ 0.01) compared with those of the control group, and the ovary weights in the POI + hUMSCs groups were obviously increased compared to those in the POI + PBS group (3.394 ± 0.1879 vs. 2.425 ± 0.1401, n = 16, *P* ＜ 0.01) (Fig. 2C).

**Fig. 3:**
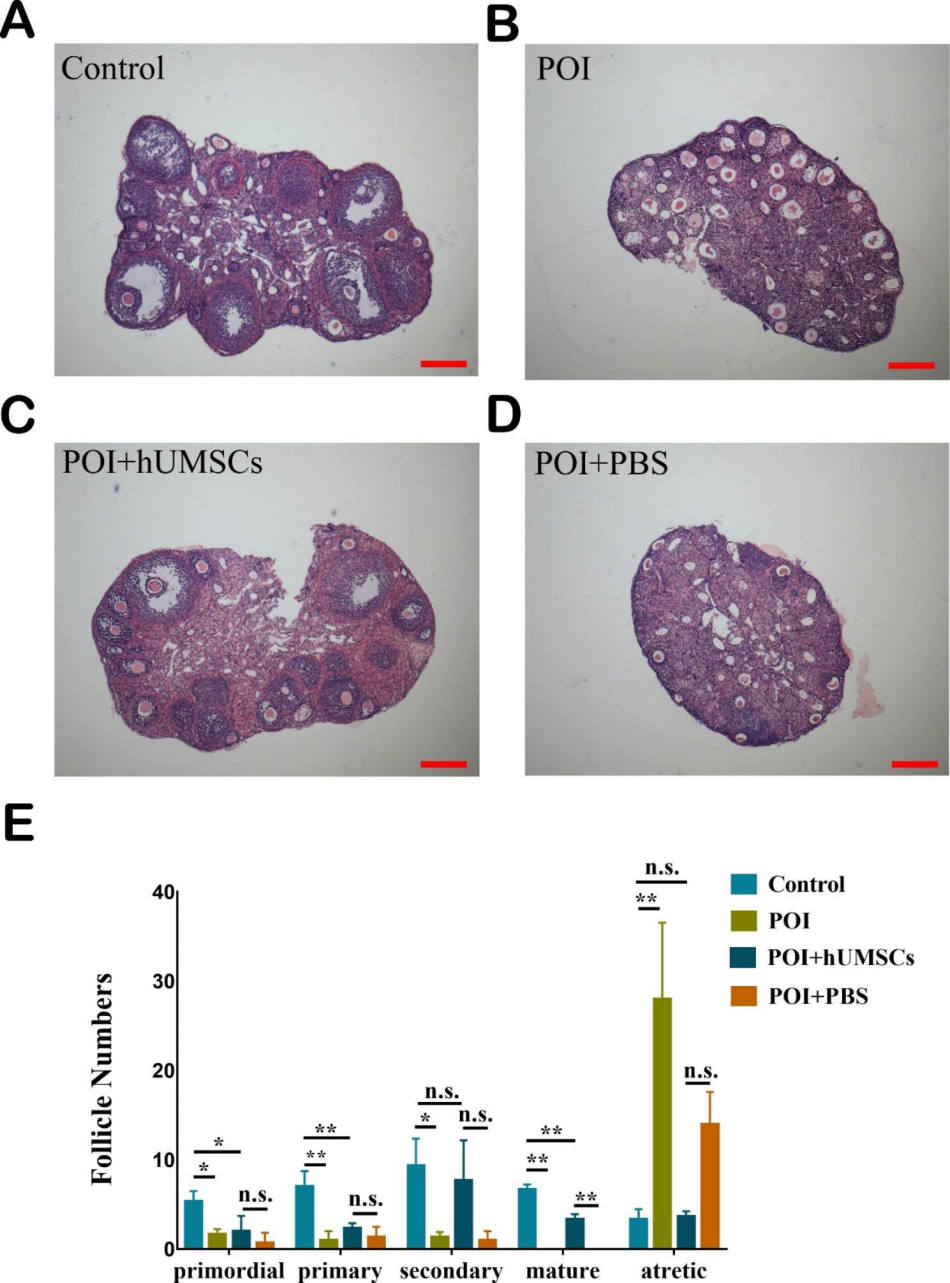
hUMSC transplantation promotes follicular development. HE staining of ovaries in **(A)** Control, **(B)** POI, **(C)** POI + hUMSCs and **(D)** POI + PBS groups. **(E)** Follicle counts of all follicle stages in Control, POI, POI + hUMSCs and POI + PBS ovaries (n = 3). Scale bar = 50 μm. *P < 0.05, **P < 0.01

We further performed HE staining to count follicle numbers in different groups. As shown in Fig. 3A, the control group showed a large accumulation of developing follicles, including primary follicles and secondary follicles, and there were many more antral follicles than in the other groups. The ovaries of POI and POI + PBS groups demonstrated fewer healthy follicles that failed to mature (Fig. 3B and D). Following hUMSCs transplantation, the number of mature follicles was significantly increased compared with that in the POI + PBS group (3.33 ± 0.33 vs. 0.00 ± 0.00, n = 3, *P* ＜ 0.01) (Fig. 3E). Overall, the results confirmed that hUMSCs transplantation restored follicle development in damaged ovaries induced by CTX + BUS treatment.

### Differential gene expression in the control, POI, POI + hUMSCs and POI + PBS groups

**Fig. 4:**
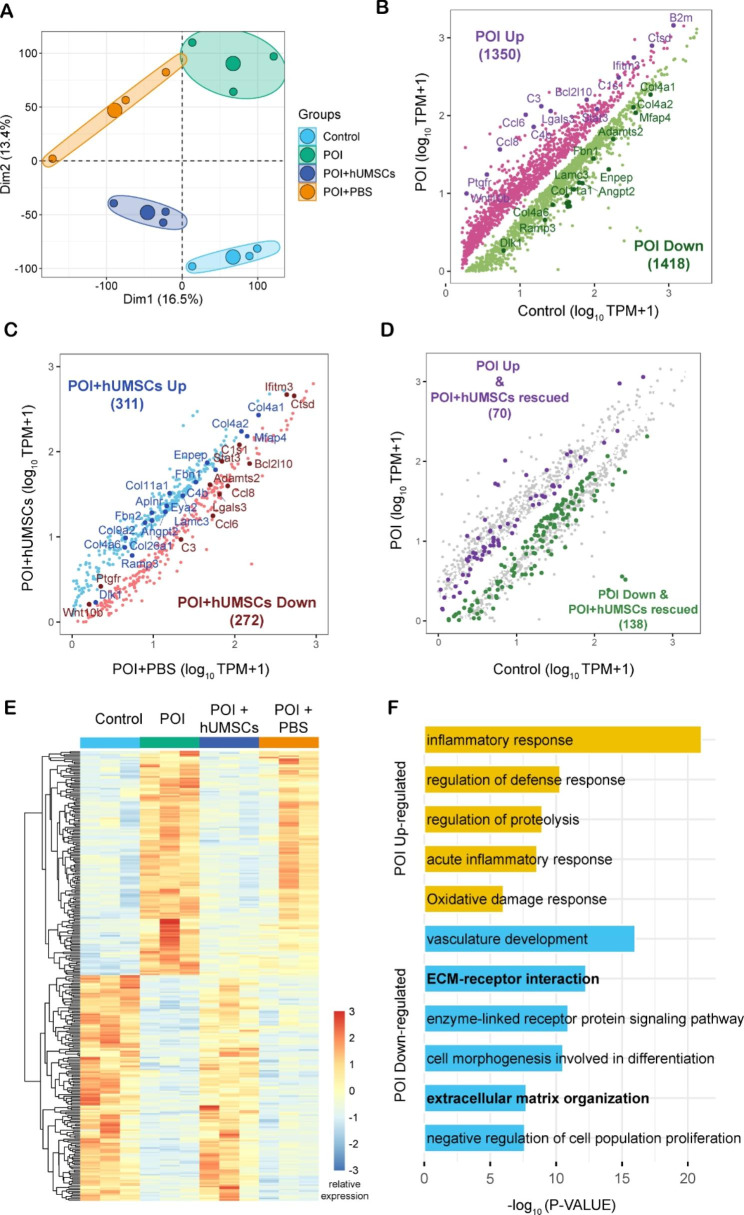
Differential gene expression analysis and DEGs functional enrichment analysis. **(A)** PCA analysis of Control, POI, POI + hUMSCs and POI + PBS groups. Scatter-plot comparison showing the DGEs between **(B)** Control and POI groups, **(C)** POI + hUMSCs and POI + PBS groups. **(D)** Scatter-plot comparison showing the POI + hUMSCs rescued DGEs between POI up-regulated and POI down-regulated genes. **(E)** The heat map showing DEGs in Control, POI, POI + hUMSCs and POI + PBS groups. **(F)** DEGs functional enrichment analysis of the POI group compared with the control group. (n = 3)

To determine differences in transcript abundance among the control, POI, POI + hUMSCs and POI + PBS groups, we performed RNA-seq on ovaries. We obtained 37–63 million reads uniquely mapped to the genome for each sample (Supplementary Table 1). The Spearman correlations exhibited highly reproducible results between biological replicates in each group (Supplementary Fig. 1). Principal component analysis (PCA) and heatmap showed that gene expression patterns were changed in the POI group but recovered to normal levels partly after hUMSCs transplantation (Fig. 4A).

As shown in Fig. 4B and E, a total of 2768 DEGs were detected between the control and POI groups, and 1350 genes were upregulated and 1418 genes were downregulated. We also compared DEGs in the POI + hUMSCs and POI + PBS groups, and found that 311 genes were upregulated and 272 genes were downregulated in POI + hUMSCs group. The results showed that Col4a1, Col4a2, Col4a6, Fbn1, Fbn2 and Lamc3 were all downregulated in POI + PBS group and their expressions were rescued in the POI + hUMSCs group. Overall, the results demonstrated that hUMSCs injection could recover ECM-related gene expression.

### GO term and KEGG pathway enrichment analysis of the DEGs

We performed DEG functional enrichment analysis using online tools in Metascape, enriched GO terms and KEGG pathways. Functional enrichment of upregulated genes involved the inflammatory response, regulation of defence response, regulation of proteolysis, acute inflammatory response and oxidative damage response. For downregulated genes, KEGG signaling pathways were related to vasculature development, ECM-receptor interaction, enzyme-linked receptor protein signaling pathway, cell morphogenesis involved in differentiation, ECM organization and negative regulation of cell population proliferation as shown in Fig. 4F. It should be noted that ECM organization is the main focus of this study.

### PPI network construction and qRT-PCR validation of ECM genes

**Fig. 5:**
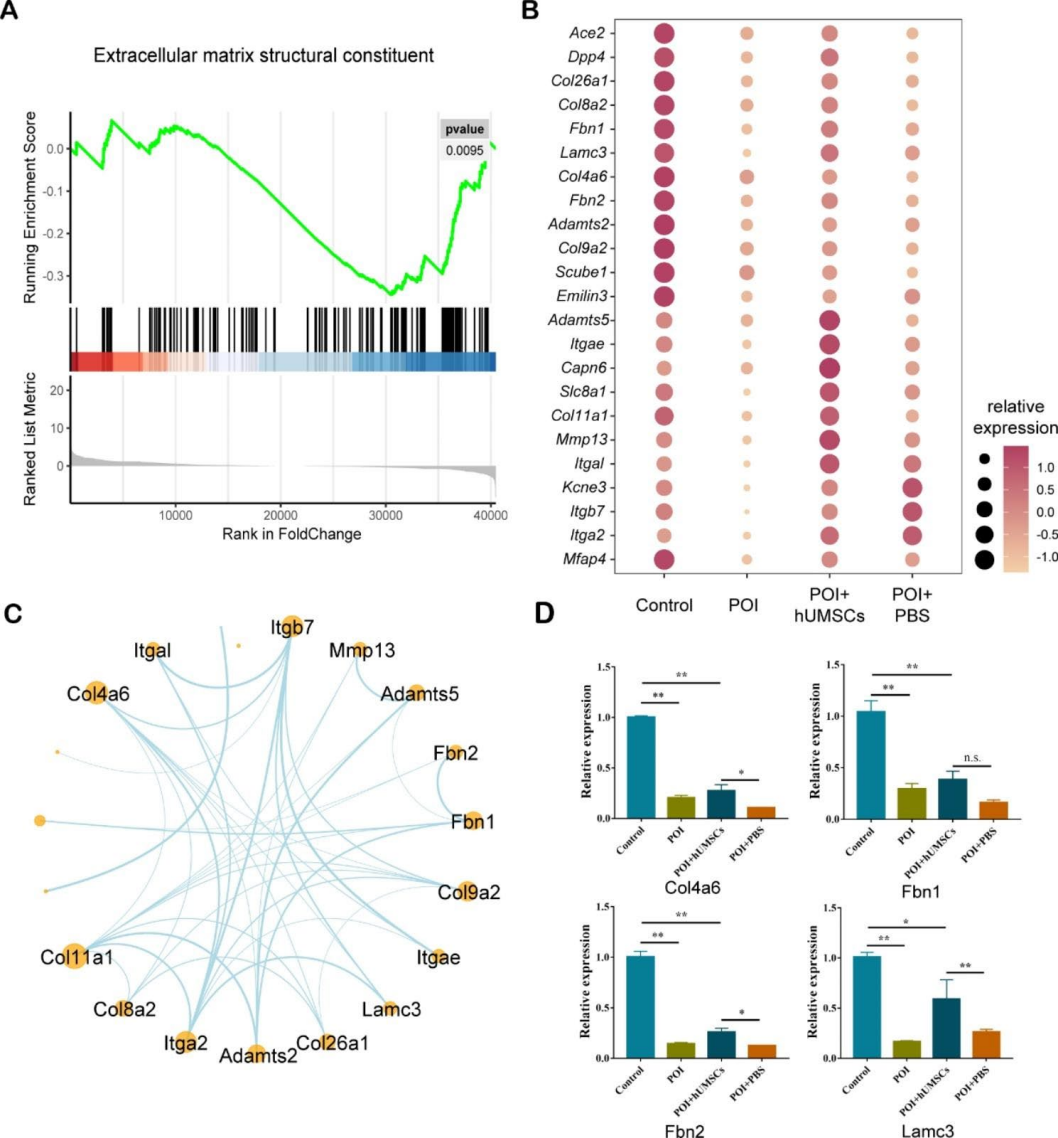
ECM-related genes GSEA analysis and PPI network construction. **(A)** GSEA analysis of extracellular matrix structural constituent. **(B)** Bubble diagram showing the enriched 23 down-regulated ECM genes in the POI group compared with the Control group. **(C)** PPI network of down-regulated ECM genes in the POI group compared with the Control group. **(D)** qRT-PCR verification of 4 down-regulated ECM genes (n = 3). *P < 0.05, **P < 0.01

We identified 23 ECM-related genes that were downregulated in the POI group and compared their expression in the control, POI, POI + hUMSCs, and POI + PBS groups (Fig. 5B). To further study the ECM structural constituent, we performed GSEA and constructed a PPI network as shown in Fig. 5A C.

To verify the transcriptome sequencing results, partial ECM-related genes that were downregulated in the POI group and rescued in the POI + hUMSCs group were selected for qRT-PCR. The qRT-PCR results for Col4a6, Fbn1, Fbn2 and Lamc3 results revealed that the expressions of all four genes were downregulated in the POI group compared with the control group and partly upregulated in the POI + hUMSCs group compared with the POI + PBS group, with the exception of Fbn1 (Fig. 5D).

### hUMSCs transplantation promoted ECM remodeling in mouse ovaries

**Fig. 6:**
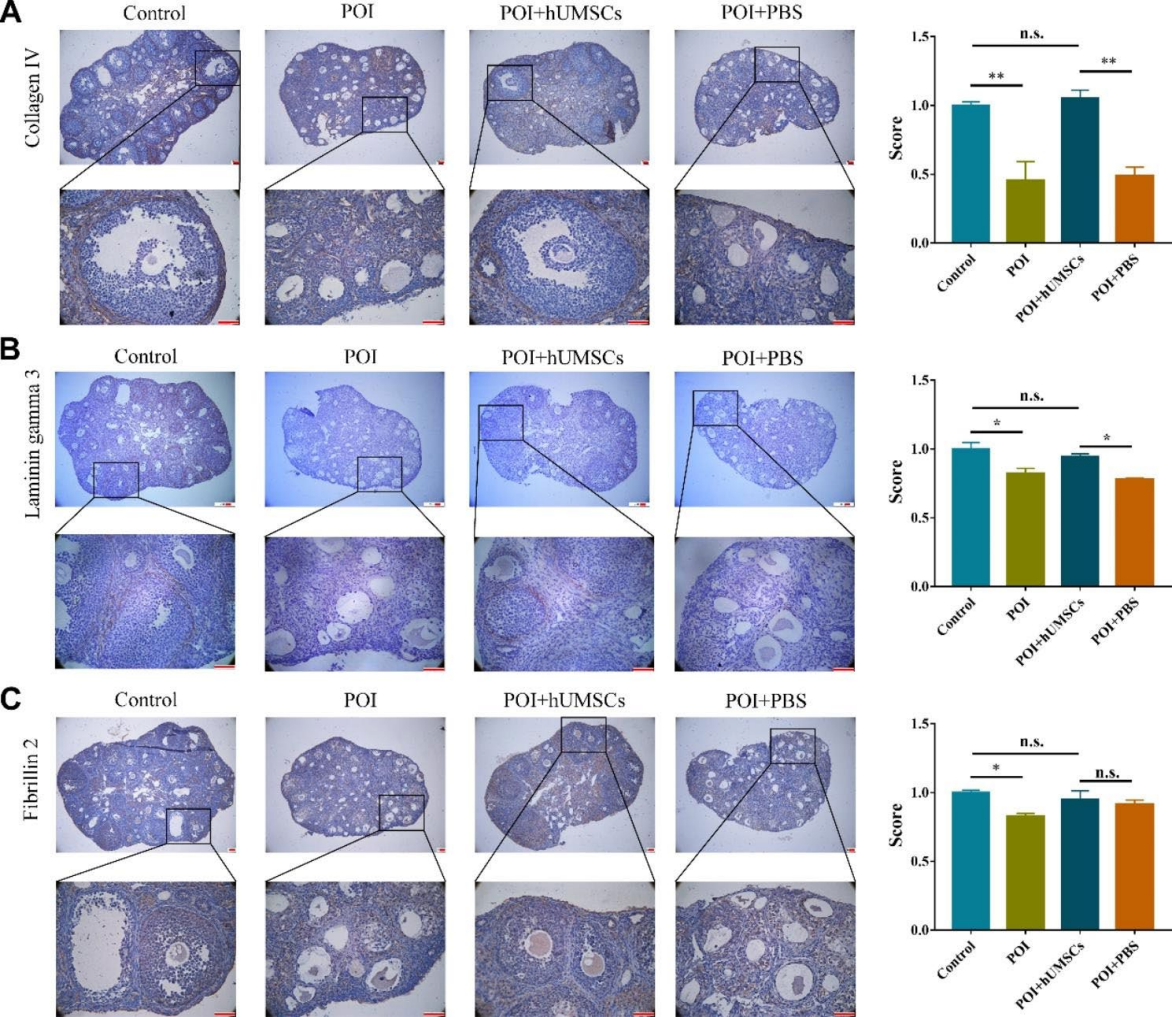
IHC of ECM proteins in ovaries. IHC and staining quantifications of **(A)** Collagen IV, **(B)** Laminin gamma 3, **(C)** Fibrillin 2 in ovaries of Control, POI, POI + hUMSCs and POI + PBS groups (n = 3). *P < 0.05, **P < 0.01

We detected the immunoexpression of collagen IV in follicles of different development stages, and the theca cell compartment showed stronger immunostaining. As shown in Fig. 6A, the control group showed obvious collagen IV expression around follicles, however the POI group showed irregular collagen IV staining in the whole ovary, and the boundaries were not clear. With the hUMSCs treatment, the expression and immunolocalization of collagen IV recovered to approximately the levels of the control group, whereas in the untreated group (injected with PBS), the expression pattern remained the same.

The localization of laminin was similar to that of collagen IV. Laminin was expressed throughout the TCs, and could clearly distinguish every grade of follicle. We selected laminin gamma 3 to perform IHC according to the transcriptome sequencing and qRT-PCR results. Our results suggested that in the POI group, laminin gamma 3 staining was weak and thus could not delineate clear boundaries, which presented a significant difference vs. the control group. Additionally, with hUMSCs transplantation, laminin gamma 3 expression recovered, but remained at extremely low levels in the PBS group (Fig. 6B).

We also enriched Fbn2 by transcriptome sequencing and qRT-PCR, and carried out IHC. However, the results showed that FBN2 was expressed in the stroma and granulosa cells but not in the theca cells (Fig. 6C).

Taken together, our studies revealed that CTX and BUS treatment led to changes in the expression and localization of collagen IV and laminin gamma 3, and hUMSCs injection partially rescued the damage induced by chemotherapeutic drugs, which could be important for normal ovulation.

## Discussion

In this study, we successfully induced mouse ovarian damage via intraperitoneal injection of CTX and BUS, and partially recovered this injury through hUMSCs transplantation. The results revealed that chemotherapy exerted negative effects on the weights of ovaries and the number of follicles at different stages, and hUMSCs injection partially rescued these negative effects and restored follicular development and gene expression partially.

Studies have shown that chemotherapy drugs may evoke ovarian damage, including follicular atresia, GC apoptosis, abnormal hormone secretion, oocyte apoptosis and fibrosis [31, 32]. Hormone replacement therapy (HRT) is one of the most common treatment modalities, but it cannot restore ovarian function from the root. Since MSCs have advantages such as easy isolation and culture and low immunogenicity, MSCs have become a hot topic in clinical and scientific research. Many studies have showed that stem cell therapy can promote follicle growth, regulate hormone secretion, suppress oocyte and GC apoptosis, inhibit cell cycle arrest and improve ovarian function [33–35].

In the study, we found it is hard to obtain mature follicles exposed to chemotherapeutic drugs. However, there exist follicles restored their development and matured finally after MSCs translation. In normal physiological conditions, most primordial follicles are in a dormant state to avoid early depletion of follicle pool. During each menstrual cycle, a small population of primordial follicles gets activated and begins to grow and develop. However, chemotherapy treatment can disturb hormone secretion and result in follicle arrest. Both LH and FSH are required for follicular maturation, it has been shown that MSCs translation could regulate hormone levels, increase the secretion of LH and FSH, and restore follicle development [36]. Our study also demonstrated that the mature follicles number was increased in POI + hUMSCs group compared with POI + PBS group (Fig. 3E).

To analyse which aspects were affected by CTX and BUS, we performed transcriptome sequencing of mouse ovaries, and further enriched gene functions and signaling pathways, therefore exploring the factors that affect follicle development and maturation. We obtained 2768 differentially expressed genes between the control and POI groups. The upregulated signaling pathways were mainly involved in the inflammatory response, oxidative damage response and other regulatory pathways, and the downregulated genes were mainly involved in signaling pathways such as ECM-receptor interaction, ECM organization and negative regulation of cell population proliferation. Changes in ECM elasticity can lead to variations in mechanical signaling. As a result, it may affect cellular communication and regulate cell physiological processes or pathological fibrosis by altering transcription factors and gene expression [37, 38]. The ECM mediates oocyte dormancy. ECM components, such as collagen IV and fibronectin, and granular cells act together to maintain the dormant state of oocytes, thus playing a role in oocyte preservation [39]. In ageing ovaries, the ECM contents were significantly reduced compared with those in younger ovaries, and the synthesis of elastin proteins in adult ovaries is also slower than that in pubertal ovaries [10, 40]. Hyaluronic acid (HA) is also a glycosaminoglycan in the ECM, and its alteration may change the structure and properties of the ECM, thereby leading to oocyte ageing and meiotic arrest [41].

Interestingly, we identified a series of ECM-related genes that were downregulated in the chemotherapeutic drug treatment groups, including Ace2, Dpp4, Col26a1, Col8a2, Fbn1, Lamc3, Col4a6, Fbn2, Col9a2, Adamts2 and other genes, and most of the above genes were upregulated after hUMSCs injection. The ECM not only acts as a supporting material, but also contains diverse receptors that mediate a variety of physiologic processes. There have been studies reporting that the ECM plays significant roles in follicular development. Therefore, we performed GSEA analysis and PPI network construction of ECM-related genes, and selected Col4a6, Fbn1, Fbn2 and Lamc3 for qRT-PCR. The results showed expressions of Col4a6, Fbn2 and Lamc3 were rescued in the POI + hUMSCs group compared with POI + PBS group (Fig. 5A-D). However, the qRT-PCR results just indicate global gene expression in the ovary tissues, and we are more interested in their locations and expressions around follicles. So, we performed IHC of Collagen IV, Laminin gamma 3 and Fibrillin 2, and the results showed Collagen IV and Laminin gamma 3 were localized around the follicles, and the immunostaining of the two proteins were decreased in the chemotherapeutic group (Fig. 6A-B). TCs distribute around the follicles [18], previous studies have shown that collagen IV and Laminin are localized at the TC region [20]. Therefore, it is reasonable to speculate that the abnormal expressions and localizations of Collagen IV and Laminin gamma 3 led to the suppression of follicular development. However, excessive ECM deposition may also result in fibrosis, and subsequently repress ovarian function. Studies have demonstrated that collage I and collage III can participate in tissue fibrosis [40, 42, 43]. Mesenchymal stem cell transplantation could reduce fibrosis levels and reverse ovarian function by inhibiting the expression collage I and collage III by decreasing the synthesis of TGFβ1 and SMAD3 [23].

It has been shown that the ECM plays essential roles in the regulation of follicular development through the Hippo and PI3K-AKT signaling pathways [13]. The Hippo signaling pathway is important for moderate follicle activation and development. When the growing follicle migrates from the cortex to the medullary regions, the Hippo signaling pathway is activated, which in turn slows the growth of follicles, avoiding early depletion of the primordial follicle pool [44]. However, overactivation of the Hippo signaling pathway may lead to excessive TCs proliferation and a disproportionate LH/FSH ratio ultimately cause polycystic ovary syndrome (PCOS) [45]. The PI3K-AKT signaling pathway functions together with the Hippo signaling pathway in follicular development, and the PI3K-AKT signaling pathway regulates primordial follicle activation and follicular development in different species [46, 47].

Previous study indicated that hUMSCs improved ovarian function by paracrine mechanism [26], the cell secreted several bioactive molecules, such as VEGF, IGF and HGF, as well as micro-RNAs and extracellular vehicles (EVs) to modified the ovarian micro-circumstances [48, 49]. Studies have shown that MSCs migrated into multiple organs after transplantation, which depended on damage degrees, blood vascular volume and chemokines regulation [50]. It has also been shown that MSCs transplantation promoted follicle growth through migrating to the ovarian hilum and medulla. The MSCs exerted their therapeutic function through the features of migration and homing [51, 52]. Regrettably, we did not perform the tracing experiment, we performed pre-experiment to confirm the influences of CTX and BUS on ovarian weights and developing follicle numbers directly. In the following experiments, we will label the MSCs with a fluorescent dye and re-construct animal models, make clear that the MSCs migrate into ovarian and then exert their effects.

Furthermore, in the present study, we focused on the whole ovary to explore DEGs at the transcriptional level in the different groups. Despite we performed IHC to verify the localizations and expressions of Collage IV and Laminin gamma 3, we did not isolate TCs to knock out col4a6 or lamc3 to explore the molecular mechanisms. We will continue to focus on this field and investigate the deeper molecular mechanisms, and we hope our findings provide a better understanding of the relationship between the ECM and follicular development.

## Conclusion

In summary, our results demonstrated that hUMSCs injection could promote follicular development and partly restore the expression of collagen IV and laminin gamma 3 which were localized around the follicular theca cell compartment. Additionally, our data revealed the differential expression patterns of transcription factors and signaling pathways among the control group, CTX and BUS treatment group, as well as hUMSCs-treated and untreated groups.

### Electronic supplementary material

Below is the link to the electronic supplementary material.

Supplementary Material 1: The spearman correlation coefficients were showed in Fig S1.

Supplementary Material 2: Alignment statistics of reads align to the reference genome were listed in Table S1.

Supplementary Material 3: qRT-PCR primers were listed in Table S2.

Supplementary Material 4: The corpus luteum numbers in each group
