# Supplementary figures and images for: hUMSC transplantation restores follicle development in ovary damaged mice via re-establish extracellular matrix (ECM) components
Source: J Ovarian Res. 2023 Aug 24;16:172. doi: 10.1186/s13048-023-01217-y (PMC10464307; doi:10.1186/s13048-023-01217-y)

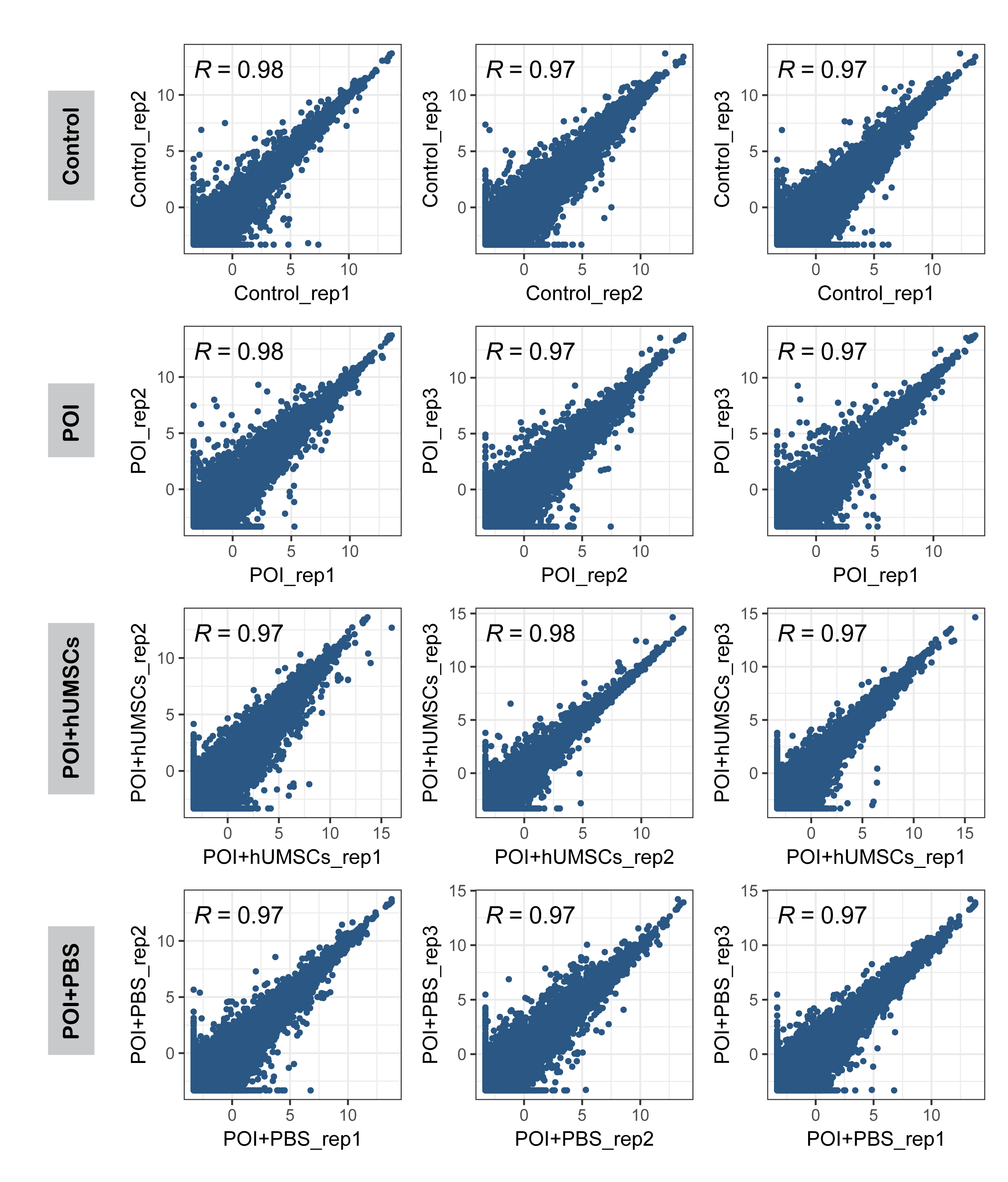

Supplement: Supplementary file 1 — Supplementary Material 1: The spearman correlation coefficients were showed in Fig S1. [file 13048_2023_1217_MOESM1_ESM.tif]
