# Supplementary material for: hUMSC transplantation restores follicle development in ovary damaged mice via re-establish extracellular matrix (ECM) components
Source: J Ovarian Res. 2023 Aug 24;16:172. doi: 10.1186/s13048-023-01217-y (PMC10464307; doi:10.1186/s13048-023-01217-y)
Supplement: Supplementary file 2 — Supplementary Material 2: Alignment statistics of reads align to the reference genome were listed in Table S1. [file 13048_2023_1217_MOESM2_ESM.docx]

**Supplementary Table 1 Alignment statistics of reads align to the reference genome.**

| **name** | **Raw reads** |  | **mapped** | **mapped** | **resds** |  |
| --- | --- | --- | --- | --- | --- | --- |
| **Control-1** | 63662896 | 60315919  (94.74%) | 3228043  (5.07%) | 57087876  (89.67%) | 38454788  (60.40%) | 18633088  (29.27%) |
| **Control-2** | 70518862 | 66818369  (94.75%) | 3485359  (4.94%) | 63333010  (89.81%) | 41914689  (59.44%) | 21418321  (30.37%) |
| **Control-3** | 44399714 | 42294463  (95.26%) | 1937682  (4.36%) | 40356781  (90.89%) | 26710461  (60.16%) | 13646320  (30.74%) |
| **POI-1** | 42975618 | 40912064  (95.20%) | 2161832  (5.03%) | 38750232  (90.17%) | 25456536  (59.23%) | 13293696  (30.93%) |
| **POI-2** | 60643100 | 57417164  (94.68%) | 3288879  (5.42%) | 54128285  (89.26%) | 35829169  (59.08%) | 18299116  (30.18%) |
| **POI-3** | 45018610 | 42710810  (94.87%) | 2319884  (5.15%) | 40390926  (89.72%) | 27221832  (60.47%) | 13169094  (29.25%) |
| **POI +hUMSCs-1** | 45977704 | 42462824  (92.36%) | 2131917  (4.64%) | 40330907  (87.72%) | 29210743  (63.53%) | 11120164  (24.19%) |
| **POI +hUMSCs-2** | 42102472 | 40037504  (95.10%) | 2152773  (5.11%) | 37884731  (89.98%) | 24985048  (59.34%) | 12899683  (30.64%) |
| **POI +hUMSCs-3** | 45569228 | 42874101  (94.09%) | 2281671  (5.01%) | 40592430  (89.08%) | 27617799  (60.61%) | 12974631  (28.47%) |
| **POI +PBS-1** | 45130374 | 42751049  (94.73%) | 2259415  (5.01%) | 40491634  (89.72%) | 27931531  (61.89%) | 12560103  (27.83%) |
| **POI +PBS-2** | 43705836 | 41499600  (94.95%) | 1929051  (4.41%) | 39570549  (90.54%) | 26251688  (60.06%) | 13318861  (30.47%) |
| **POI +PBS-3** | 56505962 | 53247058  (94.23%) | 2864811  (5.07%) | 50382247  (89.16%) | 34693421  (61.40%) | 15688826  (27.76%) |
