# Supplementary material for: hUMSC transplantation restores follicle development in ovary damaged mice via re-establish extracellular matrix (ECM) components
Source: J Ovarian Res. 2023 Aug 24;16:172. doi: 10.1186/s13048-023-01217-y (PMC10464307; doi:10.1186/s13048-023-01217-y)
Supplement: Supplementary file 3 — Supplementary Material 3: qRT-PCR primers were listed in Table S2. [file 13048_2023_1217_MOESM3_ESM.docx]

**Supplementary Table 2 Primer for qRT-PCR**

| Gene names | primers (5’-3’) | Product size (bp) |
| --- | --- | --- |
| *Gapdh* | F: CCGCCTGGAGAAACCTGCCAAG  R: CACCACCCTGTTGCTGTAGCCG | 235 |
| *Col4a6* | F: AGGCACCCTCACAAGCCATTGC  R: AAGGAGTGGCCCGGAAGTCCTC | 183 |
| *Fbn1* | F: TGGCTACCTGTGTGGCTGTCCA | 276 |
|  | R: TTCTCCACATCCCAGCTGGCGA |  |
| *Fbn2* | F: AAATGTCCGCCCGGTTTCACCC  R: GCATTCGCAGCTGAAGCTCCCA | 130 |
| *Lamc3* | F: AGGTACCAGGAGGTGCAGGCAG  R: AGCTTCTGCTCCAGGGCCTTCA | 203 |
