# Supplementary material for: hUMSC transplantation restores follicle development in ovary damaged mice via re-establish extracellular matrix (ECM) components
Source: J Ovarian Res. 2023 Aug 24;16:172. doi: 10.1186/s13048-023-01217-y (PMC10464307; doi:10.1186/s13048-023-01217-y)
Supplement: Supplementary file 4 — Supplementary Material 4: The corpus luteum numbers in each group [file 13048_2023_1217_MOESM4_ESM.docx]

**Supplementary Table 3 The corpus luteum numbers in each group**

| **Groups** | **mean ± s.d.** | **n** |
| --- | --- | --- |
| Control | 2.667±1.333 | 3 |
| POI | 0.667±0.333 | 3 |
| POI+hUMSCs | 0.667±0.333 | 3 |
| POI+PBS | 0.333±0.333 | 3 |
